# Direct and reflected self-concept show increasing similarity across adolescence: A functional neuroimaging study

**DOI:** 10.1016/j.neuropsychologia.2019.05.001

**Published:** 2019-05-08

**Authors:** Renske Van der Cruijsen, Sabine Peters, Kelly P.M. Zoetendaal, Jennifer H. Pfeifer, Eveline A. Crone

**Affiliations:** a Department of Developmental Psychology, Leiden University, the Netherlands; b Leiden Institute for Brain and Cognition, the Netherlands; c Department of Psychology, University of Oregon, USA

**Keywords:** Adolescence, Development, fMRI, Medial prefrontal cortex, Self-concept

## Abstract

In adolescence, the perceived opinions of others are important in the construction of one’s self-concept. Previous studies found involvement of medial Prefrontal Cortex (mPFC) and temporal-parietal junction (TPJ) in direct (own perspective) and reflected (perceived perspective of others) self-evaluations, but no studies to date examined differences in these processes across adolescence. In this study, 150 adolescents between 11 and 21 years old evaluated their traits from their own perspective and from the perceived perspective of peers in a fMRI session. Results showed overlapping behavioural and neural measures for direct and reflected self-evaluations, in mPFC, precuneus and right TPJ. The difference in behavioural ratings declined with age, and this pattern was mirrored by activity in the mPFC, showing a diminishing difference in activation for direct > reflected self-evaluations with increasing age. Right TPJ was engaged more strongly for reflected > direct evaluations in adolescents who were less positive about themselves, and those who showed who showed less item-by-item agreement between direct and reflected self-evaluations. Together, the results suggest that the internalization of others’ opinions in constructing a self-concept occurs on both the behavioural and neural levels across adolescence, which may aid in developing a stable self-concept.

One of the main tasks in adolescence, which is defined as the age range between 10 and 24 years (Sawyer and Azzopardi, 2018), is the development of a consistent and integrated self-concept (Harter, 2012). Theories of self-development have proposed that the internalization of perceived opinions of others about the self (reflected self-evaluations) make an important contribution to how people define themselves (direct self-evaluations) (Felson, 1985; Gecas, 1982; Mead, 1934; Quarantelli and Cooper, 1966). In adolescence, relationships with peers (including both the actual and perceived opinions of peers about the self) become increasingly important (Harter, 2012; Westenberg et al., 2004). Moreover, improving social perspective-taking skills allow adolescents to realize that there is a wider, observing audience that can evaluate the self, which may be associated with heightened self-consciousness and a rise in concerns over social evaluations (Blakemore, 2012; Somerville et al., 2013; Vartanian, 1997; Vartanian and Powlishta, 1996). The goal of this study was therefore to gain a better understanding of internalization of others’ opinions into the self-concept during adolescence. Specifically, we tested similarity between direct and reflected self-concept at different ages across adolescence. Traditionally, these studies have relied on self-report, which is inherently sensitive to bias. In this study, we take a novel perspective by examining the accompanying neural correlates of self-evaluations, which may provide additional insights above self-reports. Therefore, we used a combined behavioural and neuroimaging approach.

Neuroscientific studies on self-concept have mainly relied on paradigms in which participants evaluated sentences that described traits about the self (e.g. “I am smart”). These studies reported consistent activation in a medial regions in the prefrontal cortex (mPFC) during both direct and reflected self evaluations, which was confirmed by several meta-analyses (Denny et al., 2012; Murray et al., 2012). Developmental studies that included participants of different age groups reported that activation in mPFC is stronger in early adolescents compared to young adults in response to both direct and reflected self-evaluations (Pfeifer et al., 2007, 2009). Other studies that have used related paradigms, such as self consciousness, reported that mPFC activity peaks in adolescence when participants are being observed by others (Somerville et al., 2013; van Hoorn et al., 2016). Taken together, several studies reported an important role of the mPFC in the development of direct and reflected self-concept, but the developmental patterns of these processes are not yet well understood.

During self-evalaution tasks, the mPFC is often co-activated with several other regions, including the posterior cingulate cortex (PCC) (Pfeifer and Peake, 2012) and the temportal parietal junction (TPJ). Especially the TPJ has an important role in developmental studies that contrasted direct and reflected self evaluations (Pfeifer et al., 2009). More specifically, prior studies reported that early adolescents engage the TPJ in direct as well as reflected self-evaluations, whereas late adolescents and early adults only engage this region in reflected self-evaluations (Pfeifer et al., 2009; Veroude et al., 2014). This indicates that the TPJ has an important role in distinguishing other’s thoughts from one’s own, and in reasoning about the views of others about the self, possibly through its involvement in perspective-taking (Cabeza and Nyberg, 2000; Pfeifer et al., 2017). These previous studies compared groups of adolescents to groups of (young) adults but did not examine the full age range of adolescent development. The current study extends this research by investigating overlapping and distinct patterns of activation in response to direct and reflected self-evaluations across the broad range of adolescence. This knowledge can aid our understanding of the ungoing developments in psychological processes and their underlying neurobiological profiles related to self-concept changes at this developmental stage.

Our two main aims were (a) to determine overlapping and distinct patterns of behavioural and neural measures of direct and reflected self-concept, and (b) to test how these patterns would differ between younger and older adolescents. For this purpose, participants evaluated themselves on positive and negative traits in three domains (physical, academic, prosocial), both from their own perspective and from the perceived perspective of their peers. Most previous studies contrasted direct with reflected self-concept measures on the task level. However, similarity of the average positivity of direct and reflected self-evaluations, does not imply that individual traits are judged similarly in both conditions as well. Investigating item-by-item consistency will provide more detailed information regarding similarity in direct and reflected self-evaluations. Therefore in this study we aimed to investigate the overlap of direct and reflected self-concept across adolescence on both the task-level (average positivity of direct and reflected self-evaluations), and the item level (item-by-item correlation between direct and reflected self-evaluations).

Regarding our first aim (determine similarities and differences in behavioural and neural patterns of direct and reflected self-concept), we expected that behaviourally, direct- and reflected self-ratings (on both the task- and the item level) would be strongly related to one another. On the neural level, we expected similarity in activation especially in mPFC (Denny et al., 2012; Jankowski et al., 2014; Pfeifer et al., 2009, 2017) and TPJ (Pfeifer et al., 2009). Our second aim was to study how these patterns of overlap and distinction would differ between younger and older adolescents, and whether these would reflect a pattern indicating internalization of (perceived) opinions of others about the self. More specifically, we expected that behavioural ratings (on the task- and item level) and mPFC activation for direct and reflected self-evaluations would become more similar with age (Felson, 1985; Gecas, 1982; Harter, 2012; Quarantelli and Cooper, 1966). TPJ activation was expected to increasingly differentiate between direct and reflected self-evaluations (Pfeifer et al., 2009; Veroude et al., 2014).

An additional goal was to explore whether individual differences in positivity of direct and reflected self-evaluations were related to patterns of neural activation for direct and reflected self-concept. A previous study showed stronger ventrolateral PFC (vlPFC) activation in anxious adolescents who estimated that they were more positive about fictional peers than fictional peers would be about them (Smith et al., 2018). Interestingly, a study in adults showed a negative relationship between mPFC activation during positive self-evaluations and behavioural positivity of self-ratings, such that mPFC activation was stronger in adults who on average evaluated themselves more negatively (Pauly et al., 2013). In the current study we specifically tested whether the degree of neural overlap between direct and reflected self-evaluations in mPFC and TPJ was associated with positivity of behavioural self-concept ratings on the task level, and with item-by-item consistency on the item level.

## 1. Methods

### 1.1. Participants

This study was part of the larger Leiden Self-Concept study, of which the direct self-concept data have previously been reported (Van der Cruijsen et al., 2017). A total of 160 healthy adolescents participated. All participants were right-handed, reported normal or corrected-to-normal vision, and were not diagnosed with any neurological or psychiatric impairments. Ten participants were excluded due to excessive head movements during the fMRI scans (more than 3 mm total, n = 8), not completing the scan (n = 1), and a technical error (n = 1). Excluded participants were four 11-year-olds, two 12-year-olds, three 13-year-olds, and one 14-year old. The resulting sample of 150 participants aged between 11 and 21 years (mean age = 15.7, SD = 2.9) was used in all analyses (see Table 1 for the number of participants per age group and sex). Within this group, 95.3% (N = 143) were born in the Netherlands. All participants born outside of the Netherlands reported Dutch or European heritage. In total, 29 participants had one (N = 25) or both (N = 4) parents born outside of the Netherlands, with most of these individuals born in other European countries (58%). Participants’ parents were asked to report their gross annual family income with 6% declining to disclose. Eleven families (7.3%) reported earning less than €31.000 annually, whereas 33.3% reported a gross annual income larger than €76.000.

IQ scores were estimated with two subtests of the WISC-III or WAIS-III (Similarities and Block Design). Scores ranged between 80.0 and 137.5 (M = 110.30, SD = 11.06), and IQ did not correlate with age (r = 0.007, p = .934). All participants and both parents of minors signed informed consent before inclusion in the study. This study, titled: ‘The neural signature of self-concept development in adolescence’ (NL54510.058.16) was approved by the Medical Ethics Committee (CME) of the Leiden University Medical Centre (LUMC). Prior to the scan session, participants were screened for MRI contra-indications and self-reported psychiatric diagnoses or usage of psychotropic medication. All scans were viewed by a radiologist and no clinically relevant findings were observed.

### 1.2. Task description

**Fig. 1.**
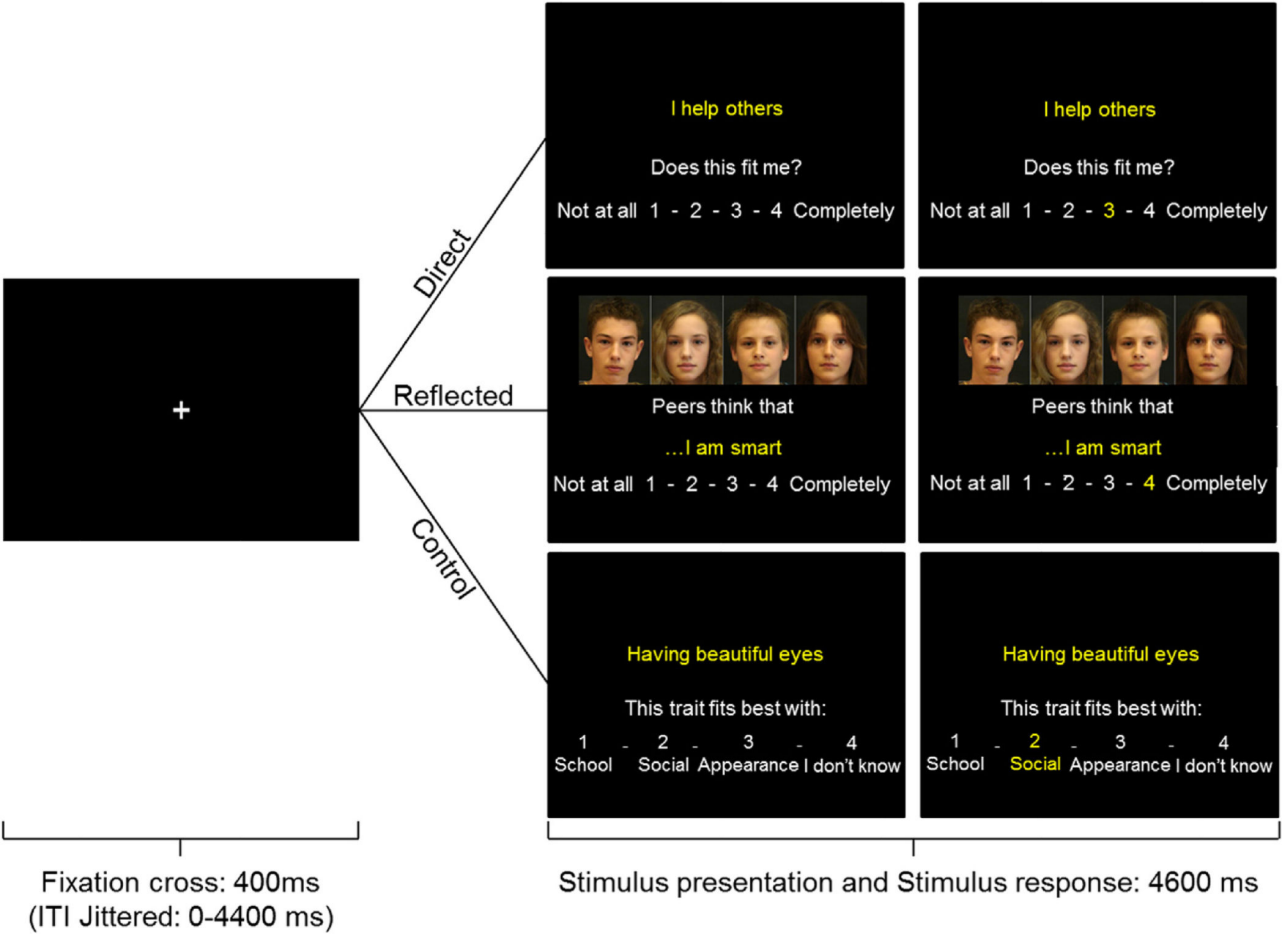
Example of a trial in the Direct, Reflected, and the Control condition. Each trial started with a black screen with a jittered duration between 0 and 4400 ms. Subsequently, a fixation cross was shown for 400 ms after which the stimulus appeared. In the Direct and Reflected conditions, participants rated on a scale of 1–4 to what extent the traits described themselves (from their own perspective or their perceived peers’ perspective, respectively). In the Control condition, participants categorized the trait sentences into one of four options. The stimulus was shown for 4600 ms. If participants responded within this timeframe, the number of their choice would turn yellow. If participants failed to respond within this timeframe, a screen with the phrase ‘Too Late!’ was shown for an additional 1000 ms after which the next trial would start. (For interpretation of the references to colour in this figure legend, the reader is referred to the Web version of this article.)

In the fMRI task, participants were presented with short sentences describing positive or negative traits in the academic, physical, or prosocial domain (Fig. 1). The task consisted of two experimental conditions (the direct self-evaluation condition, and the reflected self-evaluation condition), and a control condition. The direct self-evaluation condition and the control condition have been described before (Van der Cruijsen et al., 2017), whereas the reflected self-evaluation condition is a novel condition. In both conditions, participants responded to 60 trait sentences (e.g. ‘I am smart’, ‘I am unattractive’). In the direct self-evaluation condition, participants were asked to indicate to what extent the trait sentences applied to them on a scale of 1 (‘not at all’) to 4 (‘completely’). In the reflected self-evaluation condition, the same sentences were preceded by the words: ‘Peers think that … ‘. Morphed pictures of unknown same-aged peers were shown during these trials to remind participants to take their peers’ perspective while evaluating their traits. In both conditions, participants could indicate to what extent the traits applied to them by pressing buttons with the index to little finger of their right hand. Twenty trait sentences were shown for each domain; ten with a positive valence and ten with a negative valence. In the control condition, all response demands were the same, except that in this condition participants were asked to categorize other trait sentences according to four categories: (1) school, (2) social, (3) appearance, or (4) I don’t know. Twenty trait sentences were shown in this condition; ten with a positive valence and ten with a negative valence.

Participants completed the three conditions in separate runs, and the order of the runs was counterbalanced between participants. Within the runs, trials were presented in a pseudorandomized order with regard to domains. Each trial began with a 400 ms fixation cross. Subsequently, the stimulus was presented for 4600 ms, which consisted of the trait sentence and the response options (1–4). Within this timeframe, participants could respond to the sentence. To assure participants that their choice had been registered, the number they chose turned yellow for the remaining stimulus time. If the participant failed to respond within the 4600 ms, they were shown the phrase ‘Too late!’ for 1000 ms. These trials were modelled separately and were not included in the analysis. Too late responses occurred on 1.1% of the trials in the Direct condition, on 1.7% of the trials in the Reflected condition, and on 0.7% of trials in the control condition. The trial-order was optimized using Optseq (Dale, 1999). Additionally, OptSeq was used to add jittered intertrial intervals, which varied between 0 and 4.4 s.

We investigated our aims about behavioural similarity between direct and reflected evaluations on the task-level and on the item-level. To investigate our aims on the task-level, negative items on the direct-and the reflected self-evaluation task were reverse coded. Subsequently, scores of positive and negative items for both conditions separately were combined, resulting in an average positivity score for each condition, with higher scores indicating more positive evaluations of the self. To investigate our aims on the item-level, we created a measure of agreement by calculating item-by-item correlations for the direct and reflected task within each participant (Jennifer H Pfeifer et al., 2017). Next, we correlated both measures (the average positivity, and the item-by-item agreement) to age and neural activation.

### 1.3. fMRI data acquisition

MRI scans were acquired on a Philips 3T MRI scanner, using a standard whole-head coil. Functional scans were acquired in three runs with T2*-weighted echo-planar imaging (EPI) sequence (TR = 2200 msec, TE = 30 msec, sequential acquisition, 37 slices of 2.75 mm, FOV = 220 × 220 × 111.65 mm). To account for T1 saturation, the first two volumes were discarded. After the functional scans, a high-resolution 3D T1-FFE scan for anatomical reference was obtained (TR = shortest msec, TE = 4.6 msec, 140 slices, voxel size = 0.875 mm, FOV = 224 × 178.5 × 168 mm). Sentences were projected on a screen behind the scanner and could be seen by the participant via a mirror attached to the head coil. Head movement was restricted by placing foam inserts inside the coil.

### 1.4. fMRI preprocessing and statistical analysis

All data were analyzed using SPM8 (Wellcome Department of Cognitive Neurology, London). The functional scans were corrected for slice-timing acquisition and differences in rigid body movement. All structural and functional volumes were spatially normalized to T1 templates. The normalization algorithm used a 12-parameter affine transformation together with a nonlinear transformation involving cosine basis functions. The algorithm resampled the volumes to 3 mm cubic voxels. Templates were based on the MNI305 stereotaxic space (Cocosco et al., 1997). Functional volumes were spatially smoothed with a 6 mm FWHM isotropic Gaussian kernel.

Task effects for each participant were estimated using the general linear model in SPM8. The fMRI time series were modelled as a series of zero duration events convolved with the hemodynamic response function (HRF). Modelled events of interest for the Direct condition were “Direct-Academic-Positive”, “Direct-Academic-Negative”, “Direct-Physical-Positive”, “Direct-Physical-Negative”, “Direct-Prosocial-Positive” and “Direct-Prosocial-Negative”. The same events were modelled for the Reflected condition. For the Control condition, only one event of interest was modelled: “Control” (collapsed across domains and valences). Trials for which participants failed to respond in time were modelled as events of no interest. The events were used as covariates in a general linear model, along with a basic set of cosine functions that high-pass filtered the data. Six motion regressors were added to the model. The resulting contrast images, computed on a subject-by-subject basis, were submitted to group analyses.

To investigate our neuroimaging aims, we first performed two whole-brain one sample t-tests for the contrasts Direct > Control and Reflected > Control, followed by a conjunction analysis. The direct-evaluation trials and the reflected-evaluation trials were collapsed across domains and valences, and compared to the control trials. To test for possible age effects, we performed whole-brain regressions for the contrasts Direct > Control, Reflected > Control, and (Reflected-Control) > (Direct-Control), using age as a linear and quadratic covariate. Additionally, as reaction times decreased with age (r (148) = −0.27, p = .001), we used average reaction times as a control covariate in these analyses. For these analyses, we applied FDR cluster level correction (p < .05) at an initial uncorrected threshold of p < .001, as implemented in SPM8. All uncorrected t-maps can be found on NeuroVault (https://neurovault.org/collections/OEVTWRGL/). These maps also include analyses on gender differences, and analyses controlled for gender.

Next, we used the Marsbar ROI toolbox to create 3 ROIs, consisting of 8 mm spheres: mPFC (x = −6, y = 50, z = 4), right TPJ (x = −53, y = −59, z = 20), and left TPJ (x = 56, y = −56, z = 18). All three ROIs were based on recent meta-analyses of self-referential processing for mPFC (left-lateralized; Denny et al., 2012), and TPJ for perspective taking processes (Schurz et al., 2014). Parameter estimates were extracted from these ROIs. In all three ROIs, we first calculated the activation levels for all 1) direct and 2) reflected trials (domains and valences collapsed) versus the control trials. Next, to investigate to what extent activation for these types of self-evaluations were similar across the whole sample, we correlated the activation for Direct > Control and Reflected > Control within each ROI. Finally, we investigated whether activation elicited by both types of self-evaluations becomes more similar as adolescents get older, and with performance (average positivity and item-by-item agreement). For this purpose, we calculated the difference in activation for Reflected > Control and Direct > Control, such that positive activation indicated stronger activation for reflected self-evaluations, negative activation indicated stronger activation for direct self-evaluations, and zero activation would mean similar activation for both types of self-evaluations. We then correlated this difference score with age, and with positivity scores.

## 2. Results

### 2.1. Behavioural results

#### 2.1.1. Behavioural overlap direct and reflected self-evaluations

**Fig. 2.**
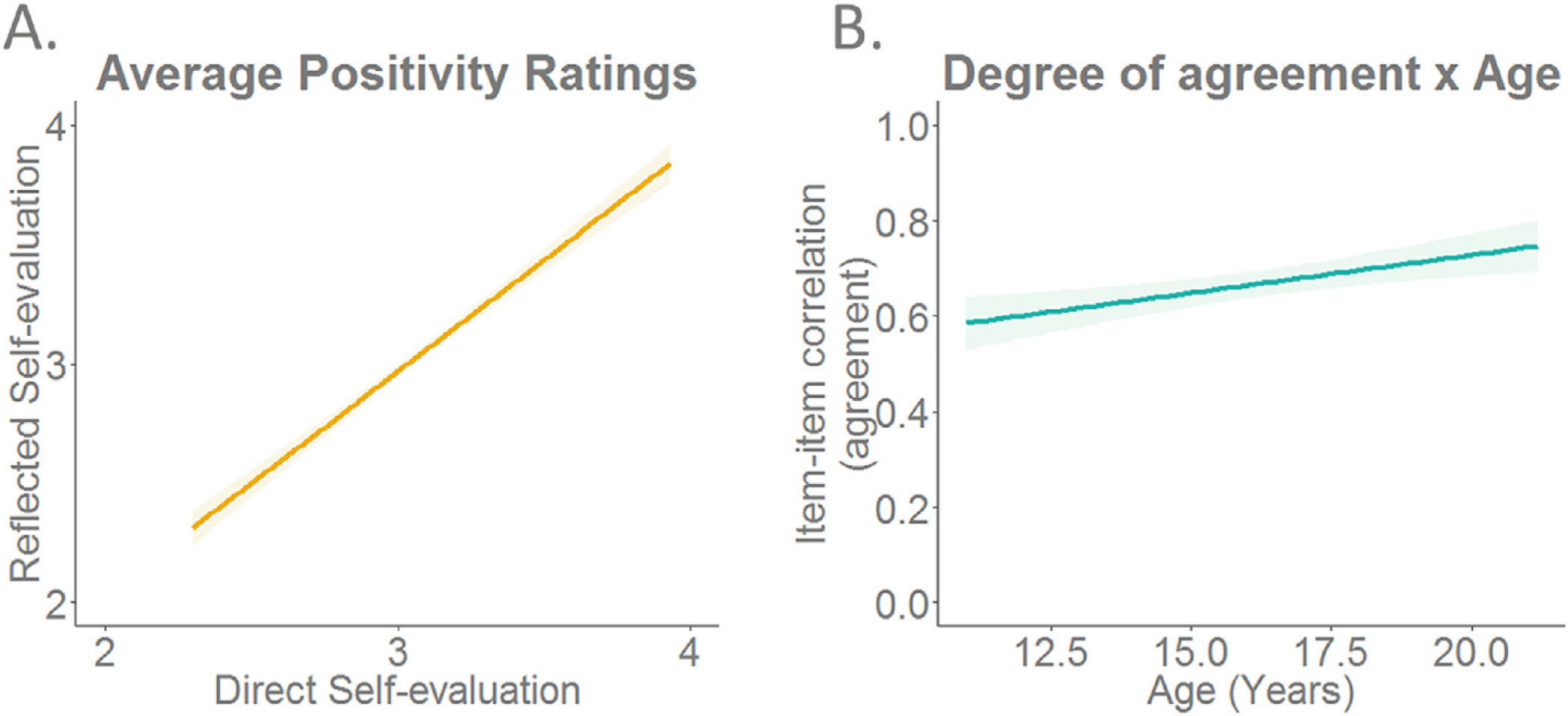
Behavioural ratings on the direct and reflected self-evaluation tasks. A. On the task-level, there is a high correlation (r = 0.87) between average positivity scores on the direct and reflected self-evaluation task. B. On the item-level, the item-by-item agreement for direct and reflected self-evaluations increases with age.

On the task-level, we compared positivity scores for direct and reflected self-evaluations across the whole sample. A paired samples *t*-test showed that participants were significantly more positive about themselves from their own perspective (M = 3.07, SD = 0.29) compared to from the perspective of their peers (M = 3.03, SD = 0.31) (t (149) = 2.8, SE = 0.01, d = 0.23 p = .006), but the correlation between average positivity scores for direct and reflected self-evaluations was high (r(148) = 0.87, p < .001) (Fig. 2a).

On the item-level, we compared positivity scores for direct and reflected self-evaluations. To do so, we created a measure of agreement by calculating item-by-item correlations for the direct and reflected task within each participant. The average item-by-item correlation was 0.66 (SD = 0.18, range: 0.08 to 0.95), which indicated generally strong item-item correlations within individuals.

Next, we investigated how the difference between ratings on both types of self-evaluations would be different for adolescents of different ages. On the task-level, a difference score was calculated for average positivity of Reflected minus Direct self-traits. This difference score showed a positive correlation with age, such that the difference score was negative in early adolescence and approached zero for older participants (r(148) = 0.22, p = .008). This indicated that the average positivity of Direct and Reflected self-evaluations becomes more similar with age (Fig. 5a and b). Separate correlations of Direct and Reflected self-evaluations with age were not significant (Direct: r(148) = −0.065, p = .43; Reflected: r(148) = 0.049, p = .55). On the item-level, we found a positive correlation of the degree of agreement on an item-by-item basis with age (r(148) = 0.269, p < .001) (Fig. 2b). This correlation demonstrated that older participants showed more agreement for direct and reflected items.

### 2.2. fMRI results

#### 2.2.1. Whole brain analyses

##### 2.2.1.1. Neural overlap direct and reflected self-evaluations.

**Fig. 3.**
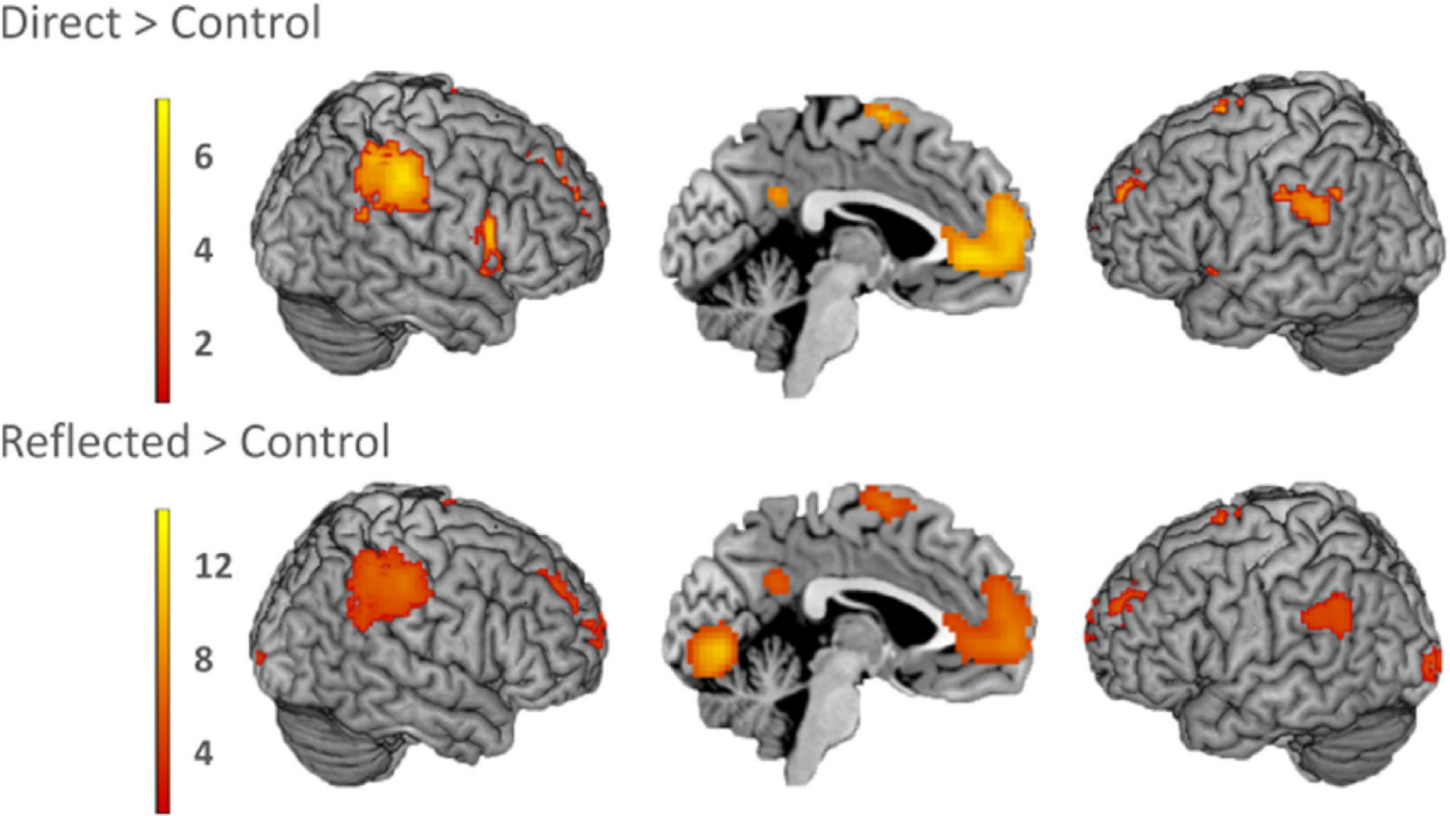
Similar activation pattern in the contrasts Direct > Control and Reflected > Control. Common activation in mPFC, bilateral supramarginal gyrus, left DLPFC, PC/PCC, and left SMA.

**Fig. 4.**
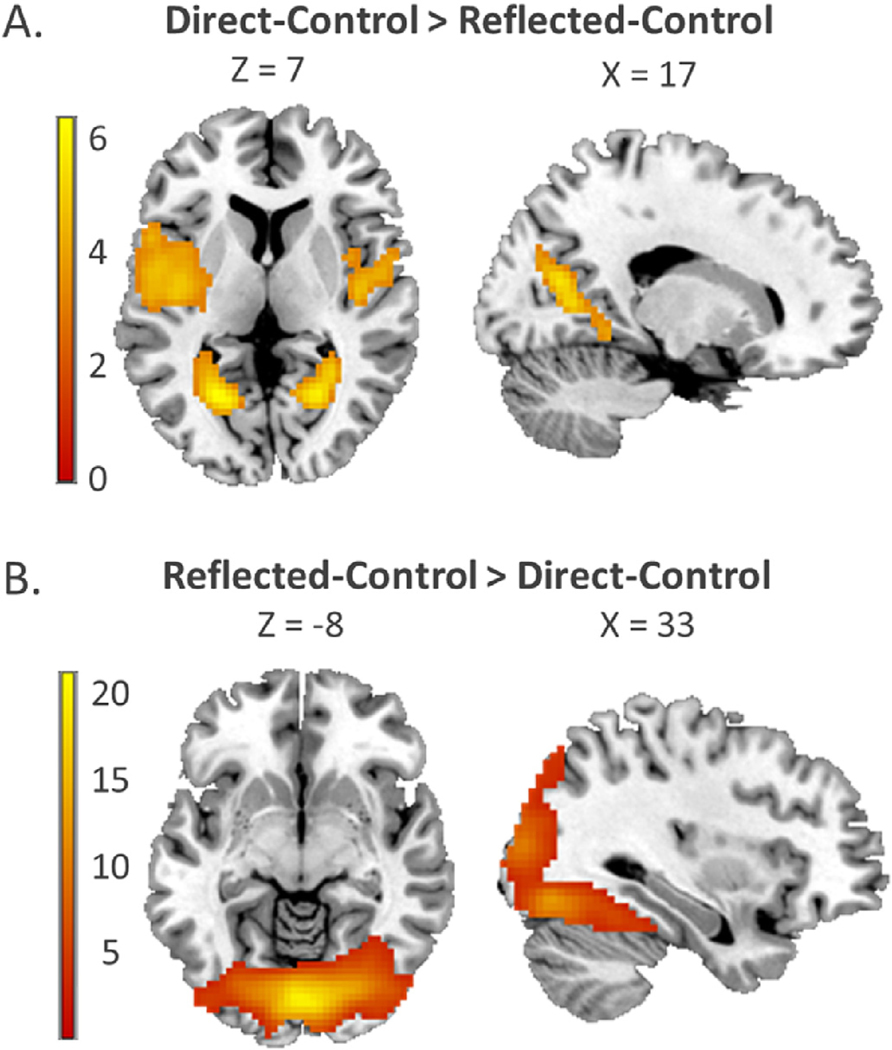
Whole-brain one-sample t-tests for the contrasts (Direct-Control) > (Reflected-Control) and (Reflected-Control) > (Direct-Control).

In order to test which brain regions were generally involved in self-evaluations, we conducted two whole-brain one-sample t-tests for Direct > Control and Reflected > Control. Both types of self-evaluation elicited a similar activation pattern, with involvement of the mPFC, bilateral supramarginal gyrus, left dorsolateral prefrontal cortex (DLPFC), precuneus/posterior cingulate cortex (PC/PCC), and left supplementary motor area (SMA) (Table 2; Fig. 3). To confirm similar activation in these regions, we performed a conjunction analysis on the Direct > Control and Reflected > Control contrasts. This analysis revealed significant activation elicited by both conditions in mPFC, right TPJ (supramarginal gyrus), bilateral DLPFC, right ventrolateral PFC (VLPFC), precuneus/PCC, and left SMA (Supplementary Table 1; Supplementary Fig. 1). To test for differences in brain activation for direct versus reflected self-evaluations, we conducted a whole-brain one-sample t-tests for the contrasts (Direct-Control) > (Reflected-Control) and (Reflected-Control) > (Direct-Control). The contrast (Direct-Control) > (Reflected-Control) resulted in bilateral calcarine gyrus extending into the hippocampus, and bilateral insula activation (Table 3a; Fig. 4a), whereas the contrast (Reflected-Control) > (Direct-Control) resulted in activation in and calcarine gyrus extending into fusiform gyrus (Table 3b; Fig. 4b). Results were similar when gender was included as a covariate of no interest. For all t-maps and additional t-maps for (Reflected-Control) > (Direct-Control) corrected for gender, see https://neurovault.org/collections/OEVTWRGL/.

**Fig. 5.**
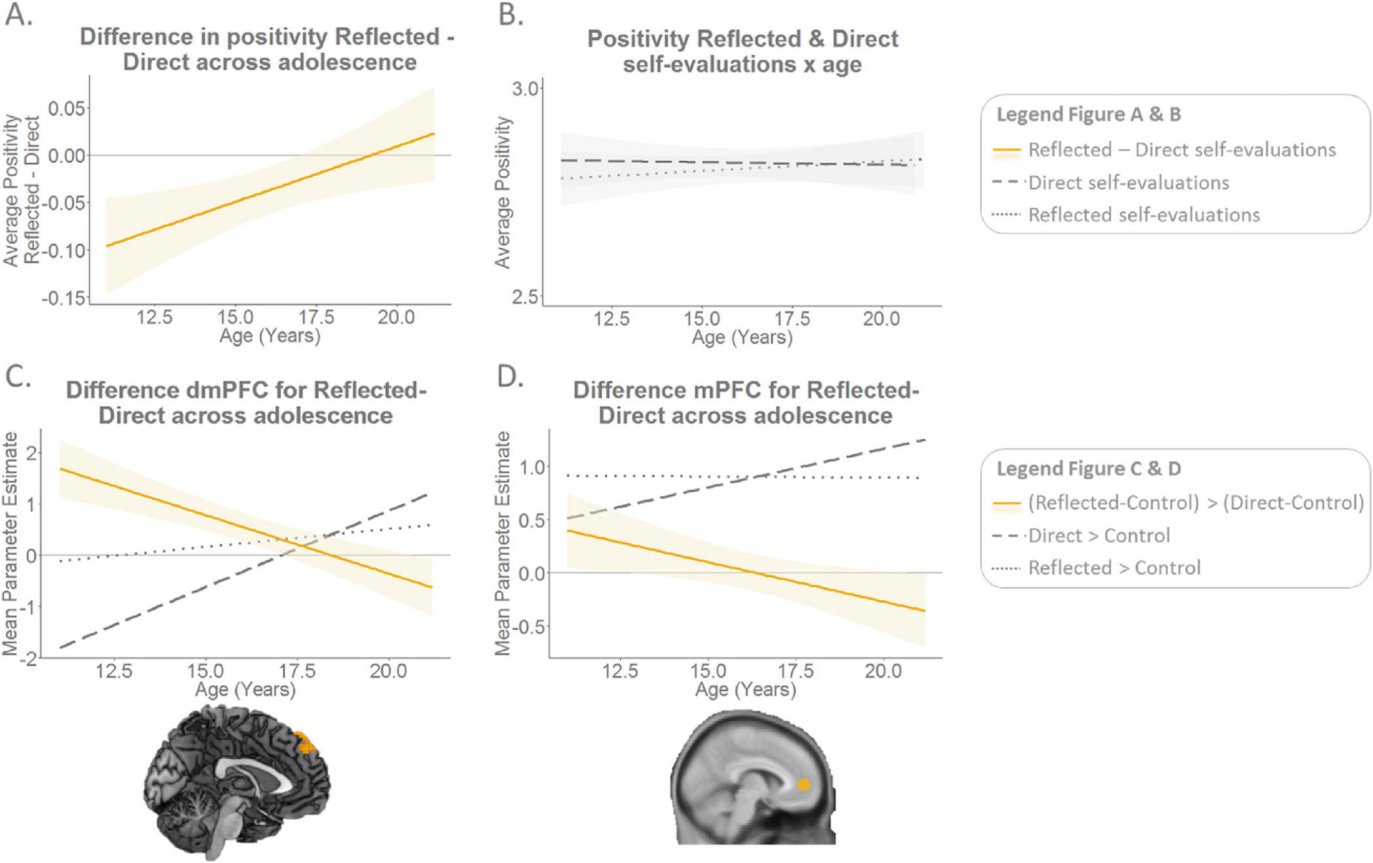
A. On the task-level, the difference in average positivity scores between direct and reflected self-evaluations decreases with age. **B.** Positivity of direct and reflected self-evaluations across age. **C.** The difference in neural activation in dmPFC (whole-brain) in response to direct and reflected self-evaluations declines with age. **D.** The difference in neural activation in the mPFC ROI in response to direct and reflected self-evaluations declines with age.

To test for possible age effects, we performed whole-brain regressions for the contrasts Direct > Control, Reflected > Control, and (Reflected-Control) > (Direct-Control), using age as a linear and quadratic covariate, and controlling for average reaction times. Two regions survived FDR-cluster correction at p < .05. First, there was a linear age effect in the dorsomedial PFC (dmPFC; x = −21, y = 50, z = 46/x = 3, y = 41, z = 58; cluster size = 77) (see Fig. 5c). Second, there was a quadratic age effect in the anterior cerebellum (x = −6, y = −46, z = −14; cluster size = 159) in the contrast (Reflected-Control) > (Direct-Control) (see Supplementary Fig. 2). Results were similar when gender was included as a covariate of no interest. For the t-maps and additional t-maps for (Reflected-Control) > (Direct-Control) x age (linear and quadratic) corrected for gender, see https://neurovault.org/collections/OEVTWRGL/.

#### 2.2.2. Region of interest analyses

Our main regions of interest, mPFC and bilateral TPJ, appeared in the above contrasts in large clusters. To ensure that we continued our analyses in independently defined regions, we constructed three 8 mm spheres based on recent meta analyses on self-concept (mPFC: Denny et al., 2012) and perspective taking (TPJ: Schurz et al., 2014). Next, parameter estimates were extracted from these ROIs (see methods section).

#### 2.2.3. Neural overlap direct and reflected self-evaluations

We investigated whether the behavioural results described above (more similarity with increasing age in direct and reflected self-concept) were mirrored in neural activation. On the task-level, we started by testing the correlations between activation for Direct > Control and Reflected > Control. These were positively correlated in all three ROIs: mPFC (r(148) = 0.79, p < .001), right TPJ (r(148) = 0.74, p < .001), and left TPJ (r(148) = 0.75, p < .001).

To investigate age-related differences in this neural overlap on the task-level, we calculated the difference scores in neural activation for Reflected > Control minus Direct > Control for each ROI and individual, and correlated these neural difference scores with age. The neural difference score in the mPFC correlated negatively with age (r (148) = 0.20, p = .015) (Fig. 5d), indicating that the difference in mPFC activation between Direct and Reflected self-ratings decreased for older adolescents. The correlation of the neural difference scores with age was not significant for left or right TPJ (both p-values > .35), neither were the separate correlations of left or right TPJ activation with direct or reflected self-evaluation with age (all p-values > .34).

#### 2.2.4. Brain-behaviour correlations

**Fig. 6.**
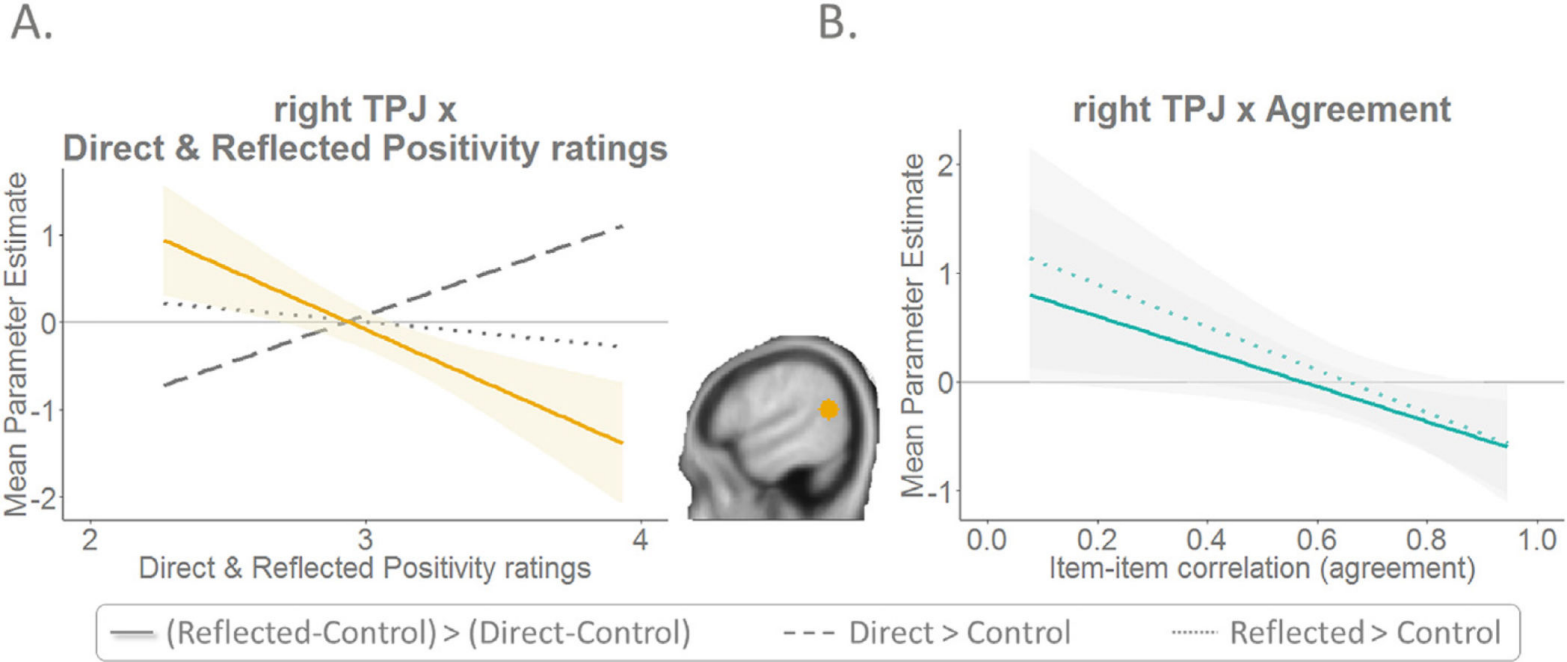
The relationship between right TPJ activation and behaviour. **A.** On the task-level, results show differential engagement of the right TPJ based on positivity of direct and reflected self-evaluations. **B.** On the item-level, results show more right TPJ engagement during reflected self-evaluations when there is less item-by-item agreement.

Last, we tested how activation of key regions in direct and reflected self-evaluations (mPFC and TPJ) were related to behaviour on the task-level. For this prupose, we correlated the neural difference scores for Reflected > Control minus Direct > Control for each ROI with the average behavioural direct and reflected positivity ratings, and with the difference between these two ratings. We found a negative correlation between the neural difference score in the right TPJ and both direct (r (148) = −0.260, p = .001) and reflected (r(148) = −0.295, p < .001) positivity-ratings (Fig. 6a). This demonstrated that adolescents who were more positive about themselves expressed relatively more right TPJ activation for direct compared to reflected self-evaluations, whereas for adolescents who are less positive about themselves, this pattern was reversed. The correlation between the neural difference scores and the average positivity ratings was not significant for left TPJ and mPFC (all p-values > .16).

To investigate the relationship between brain activation in these regions and behaviour on the item-level, we correlated the neural activation for Direct > Control, Reflected > Control, and the neural difference score as described above with the item-by-item agreement. As the item-by-item agreement correlated with age, we conducted partial correlations, corrected for age. Results showed that when there was less item-by-item agreement, there was more right TPJ activation during reflected self-evaluations, both compared to the control baseline (r(148) = −0.18, p = .029) and compared to direct self-evaluations (r (148) = −0.17, p = .040) (Fig. 6b). The correlation between the neural activations and the item-by-item agreement was not significant for left TPJ and mPFC (all p-values > .63).

## 3. Discussion

This study aimed to investigate the overlap and distinction between direct and reflected self-evaluations across adolescence (11–21-years). For this purpose, we followed two approaches: 1) determine overlapping and distinct patterns of neural correlation at the group level using conjunction and contrast analyses, and 2) exploring individual differences, by correlating the behavioural and neural measures regarding self-evaluations from one’s own and another’s perspective. Additionally, we investigated our aims on the task-level (using average positivity scores for each participant) and on the item-level (using item-by-item correlations for direct and reflected evaluations within participants). We organized the discussion along these main findings.

### 3.1. Behavioural ratings on direct and reflected self-evaluations

An important question in self-development, is the extent to which describing the self from direct and reflected perspectives is integrated (Felson, 1985; Gecas, 1982; Mead, 1934; Quarantelli and Cooper, 1966). This study addressed this question first by studying similarity in evaluation scores. Importantly, there was a strong correlation between average positivity scores (task-level) for direct and reflected self-evaluations, meaning that when someone was positive about him or herself when evaluating traits from their own perspective, they were likely to be positive about themselves from the perceived perspective of their peers as well. Similar results were found on the item-level: the average item-by-item correlation for direct and reflected self-evaluations was moderately strong. These results suggest that the measures of direct and reflected self-concept are strongly overlapping on both the task- and the item-level (Bouchey and Harter, 2005; Hergovich et al., 2002; Shrauger and Schoeneman, 1979). In addition to these overlapping ratings, the results also showed that adolescents were generally more positive about themselves when they evaluated their traits from their own perspective than when they did so from the perceived perspective of their peers.

An important goal was to investigate age-related patterns in the overlap between direct and reflected self-evaluations. The difference in average positivity between direct and reflected self-evaluations was largest in young adolescents, and declined during the adolescent period. Thus, young adolescents were more positive about themselves when they evaluated their traits from their own perspective versus from the perceived perspective of their peers, whereas older adolescents were equally positive about themselves from both perspectives. Additionally, consistent with the notion of more integration between direct and reflected self evaluations, item-by-item agreement also increased with age. Together, as hypothesized the behavioural results suggest that the process of internalizing others’ opinions in the own concept of self already starts in childhood (i.e. strong overlap between direct and reflected self-evaluations in the whole group) (Felson, 1989; Harter, 2012), but further continues throughout adolescence (i.e. stronger overlap in older adolescents).

### 3.2. Neural activation for direct and reflected self-evaluations

This study had the objective to complement findings from self-report behavioural measures with neural activity measures. The combined approach is expected to provide more insight in the underyling psychological and neural processes. We first addressed the question whether there were differences in the neural correlates of direct and reflected self-evaluations across the whole group. We found overlapping neural activation in mPFC, precuneus/PCC, bilateral DLPFC, and right TPJ (supramarginal gyrus). All these regions have often been found to be involved in evaluating one’s traits (Denny et al., 2012; Moran et al., 2010; van der Cruijsen et al., 2017). Specifically, mPFC is thought to reflect self-relevance (D’Argembeau, 2013; Denny et al., 2012), whereas TPJ activation has been related to taking the perspective of others (Schurz et al., 2014). Although activation in precuneus/PCC is consistently found in studies investigating self-concept, its function within this context is still unclear. Perhaps this region activates autobiographical memory processes (Fink et al., 1996; Northoff and Bermpohl, 2004; van der Meer et al., 2010). Alternatively, activation within this region may reflect mentalising processes such as thinking about self and others in social contexts, or comparing self to others (Cavanna and Trimble, 2006; Kedia et al., 2013; Swencionis and Fiske, 2014). DLPFC activation might reflect semantic memory retrieval (Badre and Wagner, 2007; Martinelli et al., 2012; Thompson-Schill et al., 2005), or higher order cognitive functions such as self-regulation (Coutlee and Huettel, 2012; Sanfey et al., 2003). Our results indicate that adolescents engage the above described processes when evaluating themselves from both their own and the reflected perspective. Future studies should examine the differential contribution of this network in more detail by relating the neural patterns to behavioural measures. The neural overlap for self-evaluations from one’s own and a reflected perspective is consistent with a previous study that found no differences between neural activity elicited by direct and reflected self-evaluations in adolescents, although the two conditions differed in adults (Pfeifer et al., 2009).

Despite these overlapping activation patterns, some neural differences between conditions emerged. That is, we found stronger activation in bilateral insula for direct compared to reflected self-evaluations (Ochsner et al., 2005). Previous studies showed stronger insula activation for evaluating the self compared to a public-other, but comparable insula activation for self and a close-other (Murray et al., 2012). The insula has been found to be activated in self-referential tasks with emotional components (Pfeifer and Peake, 2012), and a study in adults showed stronger insula activation in response to positive compared to negative trait evaluations (van der Cruijsen et al., 2017). Positive traits are generally rated as more self-relevant than negative traits (D’Argembeau, 2013). Hence, this finding suggests that evaluating the self from one’s own perspective might be more self-relevant, compared to evaluations of the self from a peer’s perspective (Moore et al., 2014). In addition, we found stronger activation for direct compared to reflected self-evaluations in the calcarine gyrus extending into the hippocampus, which might be due to the greater consultation of one’s own memory when evaluating the self from one’s own perspective. In contrast, for reflected compared to direct self-evaluations, we found stronger activation in the calcarine gyrus extending into the fusiform gyrus, which is possibly due to the visual differences between two runs (i.e. the presence of faces in the reflected task) (Kanwisher et al., 1997; McCarthy et al., 1997; Wilms et al., 2010).

### 3.3. Age differences in neural activation for direct and reflected self-evaluations

We investigated whether the age differences in the congruence of direct and reflected self-evaluations on the behavioural level would be mirrored on the neural level. Whole-brain results showed that the difference in dmPFC activation strength for direct versus reflected self-evaluations diminished across age. This pattern was mirrored in the ROI analysis testing a more ventral part of the mPFC. Young adolescents showed stronger (d)mPFC activation when they evaluated their traits from the perceived perspective of their peers versus their own perspective, whereas older adolescents showed similar activation levels for self-evaluations from both perspectives. This neural pattern matches the behavioural pattern in the sense that both behavioural ratings and mPFC activation for the two types of self-evaluation get increasingly similar with age.

Several hypotheses emerge from these findings. First, our results may indicate behavioural as well as neural indicators of the internalization of others’ opinions into the self-concept (Felson, 1985; Gecas, 1982; Harter, 2012; Quarantelli and Cooper, 1966). Second, the amount of value attached to the opinions of others decreases from early to late adolescence and adulthood (Knoll et al., 2015). The behavioural discrepancy in younger adolescents between direct and reflected self-evaluations may therefore also result of young adolescents caring greatly about the opinions of their peers. The stronger similarity between direct and reflected self-concept could be explained by older adolescents attaching less value to their peers’ opinions (Knoll et al., 2015). Related to this, mPFC activation has been thought to reflect personal significance of self-related stimuli (D’Argembeau, 2013). These results might indicate that reflected self-evaluations are more personally significant to early adolescents, whereas in late adolescence, direct and reflected self-evaluations are equally significant to the self. Third, across adolescence, individuals develop an increasingly clear and coherent identity and self-concept (Côté, 2009; Harter, 2012). As one’s own identity gets clearer, it is more likely that individuals assume that their peers will recognize this and will evaluate them accordingly, which would explain the increasing congruency between direct and reflected self-evaluations.

Our results showed similar TPJ activation for direct and reflected self-evaluations, and no differences in TPJ activation between adolescents of different ages. This indicates that younger as well as older adolescents recruit the TPJ when evaluating themselves from both their own and their peers’ perspective. This contradicts previous studies showing TPJ activation only for reflected self-evaluations in late adolescents and adults (Pfeifer et al., 2009; Veroude et al., 2014), but for both direct and reflected self-evaluations in early adolescents (Pfeifer et al., 2009). Possible developmental trajectories should be more thoroughly investigated in studies using longitudinal designs, preferably including early adults as well. One possible interpretation for this finding is that the TPJ is more strongly related to individual differences between adolescents, rather than to general age-patterns. This interpretation is described in more detail in the next section.

### 3.4. Relationship between neural activation and behavioural ratings

Finally, we explored individual differences in neural responses to direct and reflected self-evaluations by testing how activation of key regions in direct and reflected self-evaluations (mPFC and TPJ) were related to the behavioural average positivity scores. On the task-level, the results indicated differential engagement of the right TPJ in adolescents who were relatively more positive about themselves compared to adolescents who were relatively more negative about themselves. More specifically, adolescents who were more positive about themselves expressed relatively stronger right TPJ activation for direct versus reflected self-evaluations, whereas adolescents who were less positive about themselves showed relatively stronger right TPJ activation for reflected compared to direct self-evaluations. Two possible hypotheses emerge from these findings. First, perhaps the relatively stronger right TPJ activation for direct versus reflected self-evaluations in adolescents who are more positive about themselves is related to these adolescents taking the perspectives of others into account when engaging in direct self-evaluations (Schurz et al., 2014). Second, the relatively stronger right TPJ activation for reflected versus direct self-evaluations in adolescents who are less positive about themselves might be due to these adolescents being more concerned about the opinions of others compared to adolescents who are more positive about themselves.

On the item-level, results demonstrated that adolescents who show less item-by-item agreement, show stronger right TPJ activation during reflected self-evaluations, both compared to the control baseline and compared to direct self-evaluations. Here we replicate a recent study in Chinese young adults reporting greater engagement of the right TPJ during reflected academic self-appraisals in participants with lower agreement scores on direct and reflected self-evaluations (Pfeifer et al., 2017). Hence, the current study supports the suggestion of Pfeifer et al. (2017) that right TPJ activation facilitates the reasoning about others’ thoughts about the self (Saxe, 2010) as taking another’s perspective may be a more demanding task when others’ opinions differ from one’s own. Alternatively, stronger right TPJ activation during reflected self-evaluations in adolescents who show more incongruence between direct and reflected self-evaluations may reflect more thoughtful consideration of the opinions of one’s peers, especially when they differ from one’s own opinions. Future studies should aim to further investigate these theories.

### 3.5. Limitations and future directions

This study has characterized patterns of direct and reflected self-evaluations in adolescence using a large sample size across a large age range. Nevertheless, several limitations need to be acknowledged. One limitation lays within the addition of pictures of unfamiliar others in the reflected self-evaluation condition. These pictures were added in order to remind participants to actually take the perspective of their peers during these trials (see also van Hoorn et al., 2016). The age comparisons were not affected by this manipulation given that all participants of all ages were presented with the same stimuli. However, the addition of these pictures might confound the comparison between direct and reflected self-evaluations: processing others’ faces may induce neural activation beyond the task manipulation of evaluating others’ opinions about the self, such as neural activation for processing pictures of peers.

Although the sample size in this study is large compared to prior studies, it only included participants from age 11 onwards. In this study, the youngest adolescents showed the largest differences in behavioural ratings and neural activation between direct and reflected self-evaluations. Therefore, in order to reveal the full developmental pattern, future studies could include an even broader group of adolescents, starting at the onset of puberty (between age 8–12) (Crone and Dahl, 2012). Accordingly, it would be interesting to also investigate the effects of pubertal status (Pfeifer et al., 2013). Furthermore, there was an unexpected quadratic trend in the cerebellum (https://neurovault.org/collections/OEVTWRGL/ ). This findings was not further interpreted, but future studies with large datasets should test whether this pattern is replicable in new data sets.

Future studies should aim to apply a longitudinal design, to get more detailed knowledge on when changes in neurocognitive processes underlying direct and reflected self-evaluations occur. A longitudinal model would provide the opportunity to better study the internalization of the opinions of peers in the self-concept. Additionally, as previous studies have shown different neural mechanisms of self-evaluations in different domains and valences (Jankowski et al., 2014; van der Cruijsen et al., 2017; Van der Cruijsen et al., 2018), an interesting direction for future studies will be to investigate domain and valence differences in direct and reflected self-evaluations across adolescence. Furthermore, testing for representational patterns within the mPFC (using multivariate pattern analysis) would be a more sensitive method that could provide more specific information about the neural processes underlying direct and reflected self-evaluations across adolescence.

## 4. Conclusion

We studied the overlap of direct and reflected self-evaluations across adolescence, and whether measures of direct and reflected self-evaluations become more similar in older adolescents (both behaviourally and at the neural level). This study showed that the behavioural and neural measures for direct and reflected self-evaluations are strongly correlated and largely overlap. Importantly, the difference between behavioural evaluations from direct and reflected perspectives declined with age both on the task-level and on the item-level. This pattern was mirrored by activation in the mPFC, which was more active in the youngest adolescents for reflected than direct self-evaluations, but this difference diminished in older adolescents. These results suggest that the internalization of the opinions of others into the self-concept occurs on both the behavioural and neural level, and continues into adolescence (Felson, 1985; Gecas, 1982; Harter, 2012; Quarantelli and Cooper, 1966), indicating the importance of the adolescent period in the internalization of the perceived opinions of others about the self. These processes may aid in developing a stable self-concept. In addition, this study showed relatively stronger right TPJ activation for reflected compared to direct self-evaluations in adolescents who were less positive about themselves, possibly because these adolescents are more concerned about the opinions of others compared to adolescents who are more positive about themselves. Taken together, this study provides a comprehensive analysis of direct and reflected self-evaluations across the whole period of adolescence, and showed that two important regions that have previously been implicated in self-evaluations, mPFC and TPJ, have separable contributions to the development of self-concept. This study informs new studies which should aim to test in more detail questions related to potentially deviant patterns of self-development for example in relation to the development of internalizing disorders or burn-out (Luo et al., 2016; Slivar, 2001; Sowislo and Orth, 2013), in relation to decision-making behaviour (Pfeifer and Berkman, 2018), or questions related to interventions aimed at strengthening self-evaluations.

## Supplementary Material

Supplementary Data (Appendix A)

## Figures and Tables

**Table 1:** Number of participants per age group and sex.

Age (Years)	Females	Males	Total

**11**	8	6	14
**12**	6	8	14
**13**	7	6	13
**14**	7	7	14
**15**	10	6	16
**16**	8	7	15
**17**	9	8	17
**18**	8	7	15
**19**	8	8	16
**20**	8	7	15
**21**	1	0	1
**Total**	80	70	150

**Table 2:** Regions activated during the Direct > Control and Reflected > Control contrast.

	Region	BA	Coordinates Direct > Con FDRc = 68			Cluster Size Direct	T Direct	Coordinates Reflected > Con FDRc = 60			Cluster Size Reflected	T Reflected

Frontal/Subcortical	R Superior Medial (mPFC)	10	6	62	13	1181	7.50	6	59	13	1286	7.39
	L Superior Medial/		−6	44	1		6.85	−9	56	13		7.35
	Anterior Cingulum											
	L Superior Medial		−6	56	13		6.14	−6	59	4		7.32
	L Mid Frontal	10	−24	50	28	164	5.33	−27	47	31	163	5.96
	L Sup Frontal		−21	44	40		4.79					
	L Mid Frontal		−39	44	19		3.35					
	R Inferior Frontal	44	57	11	22	224	6.57					
	R Inferior Frontal		54	11	4		4.42					
	R Superior Temporal Pole		54	5	−2		4.37					
	R Superior Frontal							21	−1	52	88	4.02
	R Superior Frontal							24	11	61		3.94
	R Superior Frontal							18	2	64		3.84
	L Supplementary Motor	6	−6	2	67	90	6.31	−6	2	67	131	7.17
	Area (SMA)											
	L Insula	44	−42	8	4	242	4.69					
	L Superior Temporal		−48	−16	4		4.45					
	L Superior Temporal Pole		−51	5	−2		4.14					
Parietal	R Supramarginal	40	60	−28	46	458	6.61	57	−28	49	537	6.40
	R Inferior Parietal		51	−46	55		5.26	54	−43	55		5.46
	R Inferior Parietal/		51	−55	34		3.66	45	−43	49		5.34
	Angular											
	L Supramarginal/Inferior	39	−66	−40	34	82	4.25	−57	−52	37	115	4.25
	Parietal											
	L Supramarginal		−54	−31	31		3.73	−66	−46	34		3.86
	L Inferior Parietal		−57	−52	40		3.65					
	L Posterior Cingulum (PC/	23	−9	−52	28	68	4.68	−9	−52	31	121	5.93
	PCC)											
	R Posterior Cingulum		9	−49	28		4.01					
Occipital	L Mid Occipital							−27	−100	10	60	6.25
	R Lingual	18						3	−79	−2	710	14.20

Names were based on the Automatic Anatomical Labeling (AAL) atlas.

**Table 3:** Regions activated during the (Direct-Control) > (Reflected-Control) and (Reflected-Control) > (Direct-Control) contrast.

	Region	BA	Coordinates			Cluster Size	T

A. (Direct-Control) > (Reflected-Control) FDRc = 314							

Frontal/	L Insula	13	−42	−13	10	788	5.11
Subcortical	L Postcentral	1	−60	−16	19		4.94
	L Insula	1	−36	−16	19		4.84
	R Insula	13	45	−10	10	327	4.83
	R Putamen	49	30	−1	1		4.49
	R Insula	13	45	2	−5		4.35
Occipital	L Calcarine	23	−24	−61	7	352	6.35
	L Cuneus	18	−12	−79	28		5.45
	L Hippocampus		−33	−43	1		4.15
	R Calcarine	23	24	−61	7	314	5.94
	R Calcarine	18	18	−67	16		5.62
	R Cuneus	19	12	−79	31		5.38
B. (Reflected-Control) > (Direct-Control) FDRc = 4992							
Occipital	L Calcarine	18	0	−82	−5	3809	21.02
	R Superior	18	24	−94	13		11.46
	Occipital						
	L Fusiform	19	−36	−70	−17		9.82

Names were based on the Automatic Anatomical Labeling (AAL) atlas.
