# Supplementary material for: Direct and reflected self-concept show increasing similarity across adolescence: A functional neuroimaging study
Source: Neuropsychologia. Author manuscript; Available in PMC 2020 Sep 18. (PMC7500182; doi:10.1016/j.neuropsychologia.2019.05.001)
Supplement: Supplementary Data (Appendix A) [file NIHMS1572461-supplement-Supplementary_Data__Appendix_A_.docx]

**Supplementary Material**

**Suppl. Table 1.**

*Regions activated for the conjunction of Direct > Control and Reflected > Control*

|  | *Region* | *BA* | *Coordinates* | | | *Cluster Size* | *T* |
| --- | --- | --- | --- | --- | --- | --- | --- |
| Frontal/ Subcortical | R Superior Medial Frontal (mPFC) | 10 | 6 | 59 | 13 | 880 | 6.74 |
|  | L Anterior cingulum | 32 | -6 | 44 | 1 |  | 6.67 |
|  | L Superior Medial Frontal | 10 | -9 | 59 | 7 |  | 6.24 |
|  | R Inferior Operc. | 44 | 57 | 8 | 22 | 49 | 5.49 |
|  | L Mid Frontal | 10 | -24 | 50 | 28 | 132 | 5.47 |
|  | R Superior Frontal | 9 | 21 | 47 | 31 | 84 | 5.32 |
|  | R Superior Frontal | 8 | 24 | 38 | 46 |  | 3.55 |
|  | R Superior Frontal | 6 | 21 | 11 | 58 | 47 | 3.83 |
|  | R Superior Frontal | 6 | 18 | 2 | 64 |  | 3.64 |
|  | L Supplementary Motor Area (SMA) | 6 | -6 | 2 | 67 | 90 | 6.42 |
| Parietal | L Posterior Cingulum | 23 | -9 | -52 | 28 | 70 | 4.67 |
|  | R Posterior Cingulum | 23 | 9 | -49 | 28 |  | 3.98 |
|  | R Supramarginal (TPJ) | 40 | 60 | -28 | 46 | 422 | 6.27 |
|  | R Inferior Parietal |  | 51 | -46 | 55 |  | 5.05 |
|  | R Supramarginal | 39 | 60 | -49 | 31 |  | 3.48 |

Names were based on the Automatic Anatomical Labeling (AAL) atlas.

**
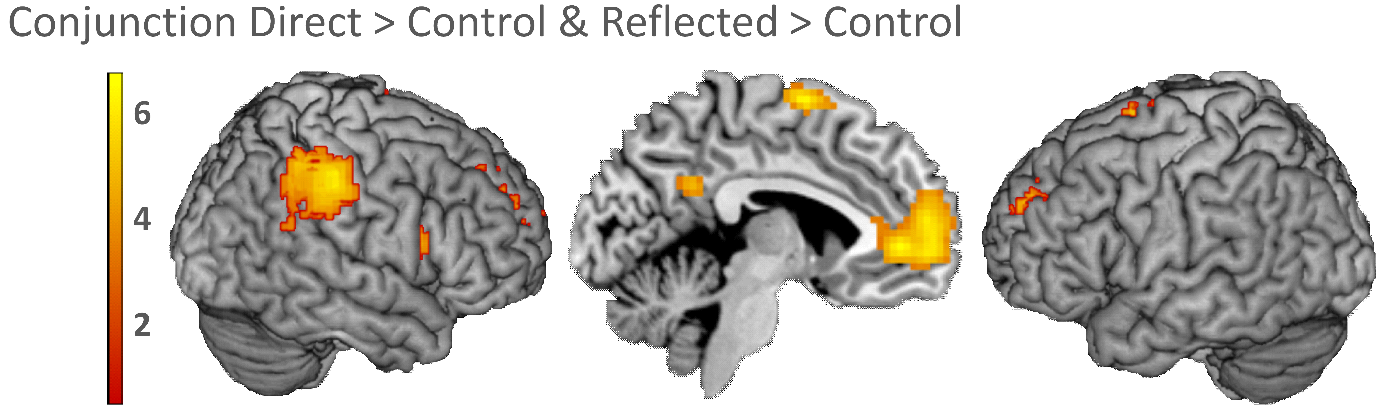
**

**Supplementary Figure 1.** The conjunction analysis of the Direct > Control and Reflected > Control contrasts showed activation in mPFC, right TPJ (supramarginal gyrus), bilateral DLPFC, right ventrolateral PFC (VLPFC), precuneus/PCC, and left SMA.

**
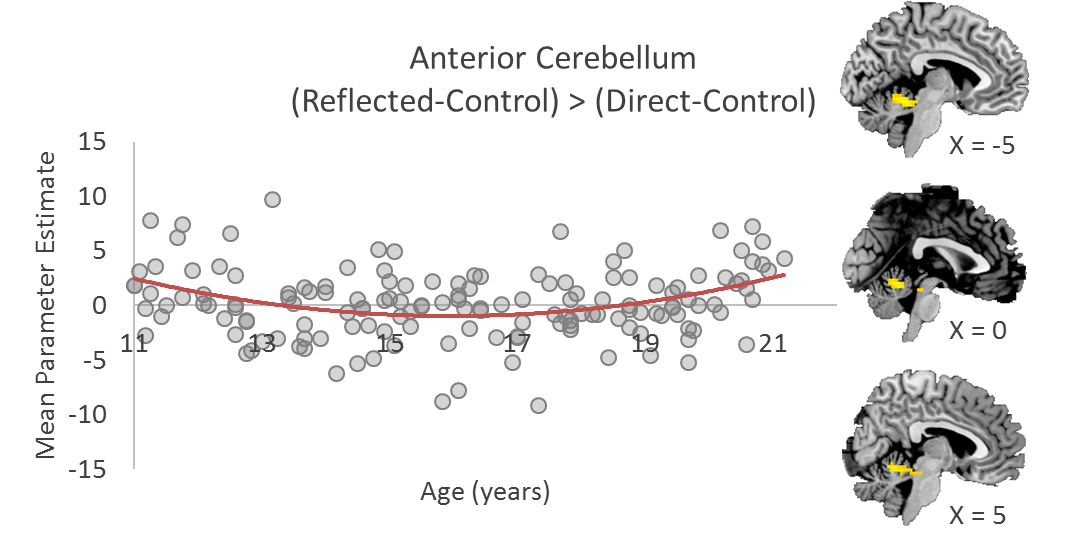
Supplementary Figure 2.** A quadratic age effect in the anterior cerebellum in the contrast (Reflected-Control) > (Direct-Control).
